# The utilization of traditional Chinese medicine in patients with dysfunctional uterine bleeding in Taiwan: a nationwide population-based study

**DOI:** 10.1186/s12906-017-1939-1

**Published:** 2017-08-29

**Authors:** Yi-Rong Lin, Mei-Yao Wu, Jen-Huai Chiang, Hung-Rong Yen, Su-Tso Yang

**Affiliations:** 1 Graduate Institute of Chinese Medicine, School of Chinese Medicine, College of Chinese Medicine, China Medical University, 91 Hsueh-Shih Rd. North District, Taichung, 404 Taiwan; 2 Department of Chinese Medicine, China Medical University Hospital, 2 Yude Rd, North District, Taichung, 404 Taiwan; 3 Management Office for Health Data, China Medical University Hospital, 2 Yude Rd, North District, Taichung, 404 Taiwan; 4 College of Medicine, China Medical University, Taichung, 404 Taiwan; 5 Research Center for Chinese Herbal Medicine, China Medical University, Taichung, 404 Taiwan; 6 Department of Biotechnology, Asia University, Taichung, 41354 Taiwan; 7 Research Center for Traditional Chinese Medicine, Department of Medical Research, China Medical University Hospital, 2 Yude Rd, North District, Taichung, 404 Taiwan; 8 Department of Radiology, China Medical University Hospital, 2 Yude Rd, North District, Taichung, 404 Taiwan

**Keywords:** Complementary and alternative medicine, Chinese herbal medicine, Dysfunctional uterine bleeding, National Health Insurance Research Database, Traditional Chinese medicine

## Abstract

**Background:**

Many patients with gynecological disorders seek traditional medicine consultations in Asian countries. This study intended to investigate the utilization of traditional Chinese medicine (TCM) in patients with dysfunctional uterine bleeding (DUB) in Taiwan.

**Methods:**

We analyzed a cohort of one million individuals randomly selected from the National Health Insurance Research Database in Taiwan. We included 46,337 subjects with newly diagnosed DUB (ICD-9-CM codes 626.8) from January 1, 1997 to December 31, 2010. The patients were categorized into TCM seekers and non-TCM seekers according to their use of TCM.

**Results:**

Among the subjects, 41,558 (89.69%) were TCM seekers and 4,779 (10.31%) were non-TCM seekers. Patients who were younger tended to be TCM seekers. Most of the patients had also taken Western medicine, especially tranexamic acid and non-steroidal anti-inflammatory drugs (NSAIDs). More than half of TCM seekers (55.41%) received combined treatment with both Chinese herbal remedies and acupuncture. The most commonly used TCM formula and single herb were Jia-Wei-Xiao-Yao-San (Bupleurum and Peony Formula) and Yi-Mu-Cao (Herba Leonuri), respectively. The core pattern of Chinese herbal medicine for DUB patients consisted of Jia-Wei-Xiao-Yao-San, Xiang-Fu (Rhizoma Cyperi), and Yi-Mu-Cao (Herba Leonuri).

**Conclusions:**

TCM use is popular among patients with DUB in Taiwan. Further pharmacological investigations and clinical trials are required to validate the efficacy and safety of these items.

## Background

Dysfunctional uterine bleeding (DUB) is defined as excessive, prolonged, frequent, and unpattern bleeding from the uterine in the absence of any structural etiology [1]. In order to standardize the terminology, diagnosis and investigations of abnormal uterine bleeding, the FIGO classification system (PALM-COEIN) was published in 2011 [2]. DUB is considered as non-structural abnormal uterine bleeding.

DUB significantly and negatively impacts the patient’s physical and social quality of life. It may put patients at risk for developing anemia, fatigue, and depression. Patients with heavy menstrual bleeding had higher hospitalization rates, emergency room visits, and outpatient visits [3]. Otherwise, heavy bleeding had significant economic implications for women because it was associated with work loss [4].

Current treatments for DUB include combined oral contraceptives, progestogens, non-steroidal anti-inflammatory drugs (NSAIDs), tranexamic acid, gonadotropin-releasing hormone analogues, danazol, and levonorgestrel-releasing intra-uterine system (LNG IUS) [5]. Endometrial ablation and hysterectomy are the surgical options for DUB. Surgical treatments are recommended in the presence of medical therapy failure, severe anemia, or other concomitant uterine pathology [6]. However, tranexamic acid not only increases the risk of thrombosis but also has side effects such as headache, anemia, and fatigue [7]. In addition to the risks of blood loss and ureteric injury, hysterectomy is not suitable for the women with fertility plan. Current conventional treatments do not fit the need of all DUB patients. Therefore, TCM therapy can provide an alternative option for these patients. TCM therapy has advantages in treating the patients with gynaecological disorders, including DUB, premenstrual syndrome, menopausal syndrome, and uterine fibroids [8, 9]. DUB is known as “flooding and spotting (Ben Lou)” in TCM literature. In TCM theory, normal menstrual cycle and fertility are regulated by the thoroughfare and controlling vessels (Chong Ren) and the essential qi of kidney. Strengthening the thoroughfare vessels, supplementing the kidney yin and yang, dissipating blood stasis, and cooling the blood to secure controlling vessels are the main principles in TCM treatments of DUB. However, a large-scale survey on the complementary TCM utilization among patients of DUB is lacking.

Therefore, this study intends to investigate TCM usage and prescription patterns for patients with DUB. We aimed to investigate the core prescription of TCM for patients with DUB and provide valuable information for TCM doctors and gynecologists. The results of this study will be useful for further research in clinical trials and pharmacological investigations in the future.

## Methods

### Data sources

The National Health Insurance (NHI) program was launched in Taiwan in 1995. It has covered more than 99% of Taiwanese residents in 2015 [10]. TCM services, including Chinese herbal medicines, acupuncture/moxibustion, and Chinese traumatology therapy, have been covered by the NHI program since 1996. The NHI administration constructed a National Health Insurance Research Database (NHIRD), which was managed by the National Health Research Institutes in Taiwan. All of the datasets were de-identified and encrypted before release for scientific research. This database contains original data including demographic characteristics, medical care facilities, outpatient and inpatient visits, visit dates, diagnostic codes, management, prescriptions and medical expenditures. The diagnostic codes were in the format of the International Classification of Diseases, Ninth Revision, Clinical Modification (ICD-9-CM).

### Study population

**Fig. 1:**
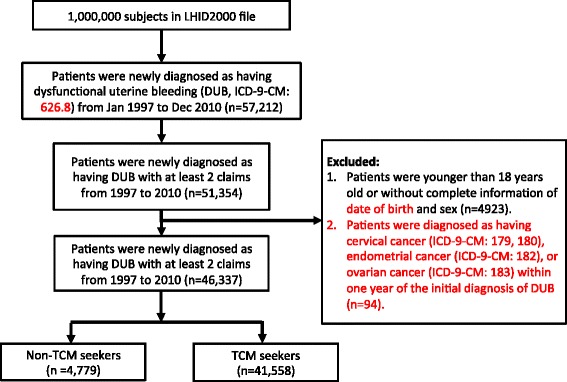
Flow recruitment chart of patients with dysfunctional uterine bleeding (DUB). We identified the newly diagnosed DUB patients from 1997 to 2000 from one million randomly selected subjects of the National health insurance research database (NHIRD) in Taiwan. Of identified 57,212 patients, 51,354 patients with at least 2 claims were included. After excluding patients according to the excluding criteria, we included 46,339 patients and separated them into TCM and non-TCM seekers according to whether they received TCM treatment or not after the initial diagnosis of DUB

A randomly selected sample with one million individuals who were enrolled in the NHI program was analyzed. Patients with newly diagnosed DUB (ICD-9-CM codes 626.8) from January 1, 1997 to December 31, 2010 were identified from the database (Fig. 1). To avoid the inclusion of patients who did not truly have DUB, we only included the patients with at least 2 claims of DUB. We excluded the patients who were less than 18 years of age, or were missing information on their sex (male and female) and date of birth. Moreover, we excluded patients who were diagnosed as having cervical cancer, endometrial cancer, or ovary cancer within one year of the initial diagnosis of DUB. TCM seekers were defined as those who visited the TCM doctors after they were diagnosed as having DUB. Non-TCM seekers were defined as patients who never visited TCM clinics after the initial diagnosis of DUB. Ultimately, 46,337 subjects were included and were divided into groups of TCM seekers (*n* = 41,558) and non-TCM seekers (*n* = 4779). This study was approved by the Research Ethics Committee of China Medical University and Hospital (CMUH104-REC2–115).

### Traditional Chinese medicine treatments

Chinese herbal formulas were listed in pin-yin name and English name. Single herbs were listed in pin-yin name, Chinese materia medica name and plant name. The TCM indications of the Chinese herbal formulas and single herbs were based on TCM theory [11, 12]. Full botanical names comply with the International Plant Names List (IPNI; http://www.ipni.org) and The Plant List (http://www.theplantlist.org/) [13]. We used a network analysis open-sourced freeware NodeXL (http://nodexl.codeplex.com/) to determine the core pattern of Chinese herbal medicine prescribed for DUB patients. As described in our previous reports [14], the thicker the line was, the more interrelated were the Chinese herbal formulas and the co-prescribed Chinese herbal formulas.

We analyzed the acupuncture and Chinese traumatology that patients received by the treatment codes in the datasets. Acupuncture includes traditional Chinese manual acupuncture, electroacupuncture, and moxibustion. Chinese traumatology indicates traditional Chinese medicine traumatology and orthopedics, which is a combination of massage, acupressure, and body manipulation.

### Statistical analysis

All statistical analyses were performed using SAS software, version 9.4 (SAS Institute Inc., Cary, NC, U.S.A.). A univariate analysis was conducted to compare the TCM seekers with the non-TCM seekers. The data analysis included descriptive statistics, including the frequency of TCM prescriptions, the patients’ demographic characteristics, indications for the prescription of TCM, and the most frequently prescribed herbal formulas and herbs for the treatment of DUB. We used t-test and chi-square test to examine the differences of numerical variables and categorical variables between two cohorts, respectively. The frequency of co-morbidities, which were the medical conditions as the reasons that DUB patients visited the clinics, between the two cohorts was compared using chi-square test. A *P*-value of <0.05 was considered statistically significant. The urbanized residence levels of all individuals were classified into four grades based on a previous study. Level 1 represents the highest urbanized level and 4 represents the lowest level [15].

## Results

Among one million beneficiaries, a total of 46,339 patients with newly diagnosed DUB between 1997 and 2010 were enrolled in this study (Fig. 1). Among these subjects, 89.69% (*n* = 41,558) visited TCM doctors for clinical consultation or treatment. Patients within the range of 18–29 years old were most likely to receive TCM treatment (Table 1). Patients who lived in highly urbanized areas preferred to use TCM. Large portion of TCM seekers also took Western medications, especially tranexamic acid and NSAIDs.

**Table 1:** Demographic characteristics of the patients with newly diagnosed dysfunctional uterine bleeding from 1997 to 2010 in Taiwan

Variable	non-TCM seekers		TCM seekers		*p* value^§^
	*n* = 4779 (10.31%)		*n* = 41,558 (89.69%)		
	n	%	n	%	
Age at baseline					<.0001
18–29	1813	37.94	19,241	46.3	
30–39	1424	29.8	12,032	28.95	
≥ 40	1542	32.27	10,285	24.75	
Mean (SD)	34.84(10.42)		32.59(9.58)		<.0001^‡^
Urbanization levels					0.0042
1 (highest)	1554	32.54	12,975	31.23	
2	1509	31.6	12,944	31.16	
3	780	16.33	7656	18.43	
4+ (lowest)	933	19.54	7966	19.18	
Conventional drug use					
Progesterone^a^	2475	51.79	26,681	64.2	<.0001
Estorgen	2291	47.94	24,531	59.03	<.0001
Combined oral contraceptives	364	7.62	4752	11.43	<.0001
Danazol	45	0.94	766	1.84	<.0001
GnRH agonists	2	0.04	18	0.04	0.99^$^
Tranexamic acid	2861	97.51	41,335	99.51	<.0001
NSAIDs	4660	97.51	41,355	99.51	<.0001
Surgery in the follow-up period^b^					0.0184
No	4230	88.51	36,288	87.32	
Yes	549	11.49	5270	12.68	

^‡^t test; ^§^Chi-square; ^$^Fisher exact test

*Abbreviations*: *TCM* traditional Chinese medicint, *NSAIDs* non-steroidal anti-inflammatory drugs

^a^Progesterone: progesterone only pills, medroxyprogesterone acetate

^b^Surgery: endometrial ablation, resection, and hysterectomy

With regard to the treatment approaches, 55.41% of the TCM seekers received combined treatment of both Chinese herbal remedies and acupuncture/traumatology, and 44.28% of patients only received prescribed Chinese herbal remedies (Table 2). Regarding the frequency of visits, 71.02% of patients visited TCM clinics for 1 to 3 times/year, while 18.48% of patients consulted TCM doctors more than 6 times/year.

**Table 2:** Distribution of different treatment types of traditional Chinese medicine received by patients with dysfunctional uterine bleeding, stratified by the number of outpatients visits

Number of TCM visits (times/per year)	Only Chinese herbal medicine	Only Acupuncture or traumatology	Combination of both treatment	Total of TCM seekers (*N* = 41,558)
	*N* = 18,401 (44.28%)	*N* = 130 (0.31%)	*N* = 23,027 (55.41%)	
	n (%)	n (%)	n (%)	n (%)
1–3	15,460 (84.02)	128 (98.46)	13,925 (60.47)	29,513 (71.02)
4–6	1﻿255 (6.82)	0	3﻿110 (13.51)	4367 (10.51)
>6	1689 (9.16)	2 (1.54)	5992 (26.02)	7678 (18.48)

*Abbreviation*: *TCM* traditional Chinese medicine

Acupuncture includes traditional Chinese manual acupuncture, electroacupuncture, and moxibustion. Traumatology indicates traditional Chinese medicine traumatology and orthopedics, which is a combination of massage, acupressure, and body manipulation

We compared the frequency of different diseases, included DUB-related and unrelated co-morbidities, between non-TCM and TCM seekers (Table 3). TCM seekers had high frequency of anemia, menopausal syndrome, and female infertility. Moreover, TCM seekers also had higher frequency in psychological symptoms such as depression, insomnia, or sleep disturbance. High incidence of vertigo/dizziness, migraine/headache, digestive disorders, and upper respiratory infection in TCM seekers were also demonstrated.

**Table 3:** Frequency of different diseases in patients with dysfunctional uterine bleeding

Disease (ICD-9-CM)	Non-TCM seekers		TCM seekers		*p* value*
	Frequency	%	Frequency	%	
DUB related					
Anemia (280.9, 281.8, 285.9)	799	16.72	9583	23.06	<.0001
Menopausal syndrome (627)	746	15.61	9071	21.83	<.0001
Female infertility (628)	311	6.51	5807	13.97	<.0001
Complications of pregnancy, child birth and the puerperium (630–676)	1761	36.85	18,785	45.20	<.0001
DUB unrelated					
Vertigo, dizziness, Meniere’s Syndrome (386, 780.4, 780.7)	1813	37.94	25,138	60.49	<.0001
Depression, insomnia, sleep disorders (300, 311, 307.4, 780.5)	1449	30.32	25,976	62.51	<.0001
Migraine and headache (346, 784.0)	1753	36.68	27,494	66.16	<.0001
Upper respiratory tract infection (460–465, 784.1)	4475	93.64	41,111	98.92	<.0001
Digestive disorders (536, 564, 787.7)	2557	53.50	33,740	81.19	<.0001

*Chi-square test

*Abbreviation*: *TCM* traditional Chinese medicine, *DUB* dysfunctional uterine bleeding

**Fig. 2:**
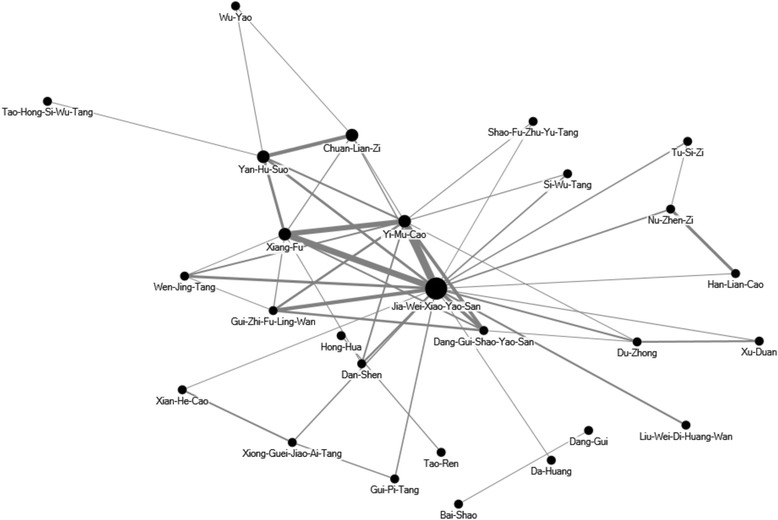
The core pattern of Chinese herbal medicine prescriptions for DUB patients. The top 50 herbal formulas and single herbs for DUB patients were assessed. The core pattern of the Chinese herbal medicine prescriptions was the combination of Jia-Wei-Xiao-Yao-San, Xiang-Fu, and Yi-Mu-Cao

To identify the prescription patterns, we further analyzed the Chinese herbal formulas prescribed by TCM doctors. The most commonly used TCM formula and single herb were Jia-Wei-Xiao-Yao-San (Bupleurum and Peony Formula) and Yi-Mu-Cao (Herba Leonuri), respectively (Table 4 and Table 5). The core patterns of Chinese formulas and herbs prescribed for DUB patients were examined in the network analysis. The core pattern and the most frequently used combinations of formulas and single herbs consisted of Jia-Wei-Xiao-Yao-San, Xiang-Fu (Rhizoma Cyperi), and Yi-Mu-Cao (Herba Leonuri) (Fig. 2).

**Table 4:** The top ten most commonly prescribed herbs for patients with dysfunctional uterine bleeding

Pin-yin name	Chinese materia medica name	Botanical name	Indication for TCM syndrome		Frequency of prescription, times (%)	Average daily dose (g)
			DUB related	DUB unrelated		
Yi-Mu-Cao	Herba Leonuri	Leonurus heterophyllus Sweet	Menstrual irregularities due to blood stasis with edema		4341 (0.08)	6.9
Xiang-Fu	Rhizoma Cyperi	*Cyperus rotundus* L	Irregular menstruation because of liver qi stagnation	Breast distention	2802 (0.05)	6.5
Yan-Hu-Suo	Rhizoma Corydalis	Corydalis yanhusuo W. T. Wang	Irregular menstruation because of liver qi stagnation	Pain relief	1742 (0.03)	6.6
Du-Zhong	Cortex Eucommiae Ulmoidis	Eucommia ulmoides Oliv.	Deficiency in liver and kidney	Weakness of muscles, tendons, and bones	1617 (0.03)	7.6
Xian-He-Cao	Herba Agrimoniae	*Agrimonia eupatoria* L. var. pilosa Mak		Excessive bleeding	1504 (0.03)	6.8
Dan-Shen	Radix Salviae Miltiorrhizae	Salvia miltiorrhiza Bge.	Irregular menstruation because of blood and qi stagnation	Pain relief	1477 (0.03)	7.9
Nu-Zhen-Zi	Fructus Ligustri Lucidi	*Ligustrum lucidum*	Yin deficiency in liver and kidney		1213 (0.02)	7.2
Xu-Duan	Radix Dipsaci	Dipsacus asperoides, C. Y.Chent et TM Ai	Deficiency in liver and kidney	Weakness of muscles, tendons, and bones	1191 (0.02)	7.2
Tu-Si-Zi	Semen Cuscutae Chinensis	Cuscuta chinensis Lam.	Yin deficiency in liver and kidney, infertility		1133 (0.02)	7.0
Chuan-Lian-Zi	Fructus Meliae Toosendan	*Melia azedarach* L. sub. Var. Toosendan Makino	Liver qi stagnation	Pain relief	1028 (0.02)	6.7

*Abbreviation*: *TCM* traditional Chinese medicine, *DUB* dysfunctional uterine bleeding

Frequency; the used times of the specific herb; %: the used times of the specific herb over the used times of all herbs for DUB patients

**Table 5:** The top ten most commonly prescribed formulas for patients with dysfunctional uterine bleeding

Pin-yin name	English name	Constitutions			Indication for TCM syndrome		Frequency of prescription, times (%)	Average daily dose (g)
		Pin-yin name	Chinese material medica name	Botanical name	DUB related	DUB unrelated		
Jia-Wei-Xiao-Yao-San	Bupleurum and Peony Formula	Dang-Gui	Radix Angelicae Sinensis	Angelica sinensis (Oliv.) Diels	Irregular menstruation because of spleen qi deficiency and liver blood deficiency with heat, liver qi stagnation	Irritability, abdominal pain, depression	7654 (0.14)	7.3
		Fu-Ling	Poria,	Poria cocos (Schw.) Wolf				
		Zhi-Zi	Fructus Gardeniae	*Gardenia jasminoides* J.Ellis				
		Bo-He	Herba Menthae Haplocalycis	Mentha haplocalyx Briq.Field				
		Bai-Shao	Radix Paeoniae Alba	*Paeonia lactiflora* Pall				
		Chai-Hu	Radix Bupleuri	Bupleurum chinense DC.				
		Gan-Cao	Radix Glycyrrhizae	Glycyrrhiza uralensis Fisch				
		Bai-Zhu	Rhizoma Atractylodis Macrocephalae	Atractylis macrocephala Koidz				
		Mu-Dan-Pi	Cortex Moutan Radicis	*Paeonia suffruticosa* Andr.				
		Wei-Jiang	Rhizoma Zingiberis officinales	*Zingiber officinale* Rosc.				
Dang-Gui-Shao-Yao-San	Dang Gui and Peony Powder	Dang-Gui	Radix Angelicae Sinensis	Angelica sinensis (Oliv.) Diels	Liver blood deficiency	Abdominal pain, dizziness, edema with inhibited urination	3147 (0.06)	6.6
		Fu-Ling	Poria	Poria cocos (Schw.) Wolf				
		Bai-Shao	Radix Paeoniae Alba	Paeonia lactiflora Pall.				
		Bai-Zhu	Rhizoma Atractylodis Macrocephalae	Atractylodes macrocephala Koidz				
		Chuan-Xiong	Rhizoma Chuanxiong	Ligusticum chuanxiong Hort.				
		Ze-Xie	Rhizoma Alismatis	*Alisma plantago-aquatica* L.				
Gui-Zhi- Fu-Ling-Wan	Cinnamon and Poria Pills	Gui-Zhi	Ramulus Cinnamomi Cassiae	*Cinnamomum cassia* Blume	Blood stasis in pelvic cavity	Lower abdominal pain	2841 (0.05)	6.6
		Fu-Ling	Poria	Poria cocos (Schw.) Wolf				
		Mu-Dan-Pi	Cortex Moutan Radicis	Paeonia suffruticosa Andr.				
		Chi-Shao	Radix Paeoniae Lactiforae	Paeonia lactiflora Pall.				
		Tao-Ren	Semen Persicae	*Prunus persica* (L.) Batsch.				
Wen-Jing-Tang	Flow warming decoction	Wu-Zhu-Yu	Fructus Evodiae Rutaecarpae	Evodia rutaecarpa (Juss.) Benth.	Blood stasis		2345 (0.04)	6.7
		Gui-Zhi	Ramulus Cinnamomi Cassiae	Cinnamomum cassia Blume				
		Dang-Gui	Raidx Angelicae Sinensis	Angelica sinensis (Oliv.) Diels				
		Chuan-Xiong	Radix Chuanxiong	Ligusticum chuanxiong Hort.				
		Bai-Shao	Radix Paeoniae Alba	Paeonia lactiflora Pall.				
		E-Jiao	Colla Corii Asini	*Equus asinus* L.				
		Mai-Men-Dong	Tuber Ophiopogonis Japonici	Ophiopogon japonicas (Thunb.) Ker_Gawl				
		Mu-Dan-Pi	Cortex Moutan Radicis	Paeonia suffruticosa Andr.				
		Ren-Shen	Radix Ginseng	*Panax ginseng* C. A. Mey				
		Gan-Cao	Radix Glycyrrhizae	Glycyrrhiza uralensis Fisch				
		Sheng-Jiang	Rhizoma Zingiberis officinales	Zingiber officinale Rosc.				
		Ban-Xia	Rhizoma Pinelliae Ternatae	Pinellia ternate (Thunb.) Breit				
Xiong-Guei-Jiao-Ai-Tang	Decoction of Donkey-Skin Glue and Artemisia	Chuan-Xiong	Rhizoma Chuanxiong	Ligusticum chuanxiong Hort.	Blood deficiency	Prevents miscarriage	3147 (0.06)	6.4
		Dang-Gui	Raidx Angelicae Sinensis	Angelica sinensis (Oliv.) Diels				
		E-Jiao	Colla Corii Asini	Equus asinus L.				
		Gan-Cao	Radix Glycyrrhizae	Glycyrrhiza uralensis Fisch				
		Shu-Di-Huang	Radix Rehmanniae	Rehmannia glutinosa Libosch				
		Bai-Shao	Radix Paeoniae Alba	Paeonia lactiflora Pall				
		Ai-Ye	Folium Artemnisiae Argyi	Artemisia argyi Levl. et Vant.				
Gui-Pi-Tang	Restore the Spleen Decoction	Ren-Shen	Radix Ginseng	Panax ginseng C. A. Mey	Blood deficiency, vaginal spotting because of qi deficiency	Diarrhea because of qi deficiency in spleen	1812 (0.03)	6.7
		Long-Yan-Rou	Arillus Euphoriae Longanae	Dimocarpus longans Lour.				
		Huang-Qi	Radix Astragali	Astragalus henryi Oliv.				
		Gan-Cao	Radix Glycyrrhizae	Glycyrrhiza uralensis Fisch				
		Bai-Zhu	Rhizoma Atractylodis Macrocephalae	Atractylis macrocephala Koidz				
		Fu-Ling	Poria	Poria cocos (Schw.) Wolf				
		Mu-Xiang	Radix Aucklandiae	Aucklandia lappa DC.				
		Dang-Gui	Raidx Angelicae Sinensis	Angelica sinensis (Oliv.) Diels				
		Suan-Zao-Ran	Semen Zizyphi Spinosae	Ziziphus jujube var. Spinosa (Bunge) Hu ex H. F. ﻿Chow﻿				
		Yuan-Zhi	Radix Polygalae Tenuifoliae	Polygala tenuifolia Willd.				
		Sheng-Jiang	Radix Zingiberis officinalis	Zingiber officinale Rosc.				
		Da-Zao	Fructus Zizyphi Jujube					
				Ziziphus jujuba Mill.				
Si-Wu-Tang	Four Substances Decoction	Shu-Di-Huang	Radix Rehmanniae	Rehmannia glutinosa Libosch	Blood deficiency		1554 (0.03)	6.9
		Bai-Shao	Radix Paeoniae Alba	Paeonia lactiflora Pall				
		Dang-Gui	Radix Angelicae Sinensis	Angelica sinensis (Oliv.) Diels				
		Chuan-Xiong	Rhizoma Chuanxiong	Ligusticum chuanxiong Hort.				
Bu-Zhong-Yi-Qi-Tang	Tonify the Middle and Augment the Qi Decoction	Huang-Qi	Radix Astragali	Astragalus henryi Oliv.	Vaginal spotting because of qi deficiency	Weakness because of qi deficiency	1317 (0.02)	6.7
		Ran-Shen	Radix Ginseng	Panax ginseng C. A. Mey				
		Bai-Zhu	Rhizoma Atractylodis Macrocephalae	Atractylis macrocephala Koidz				
		Gan-Cao	Radix Glycyrrhizae	Glycyrrhiza uralensis Fisch				
		Dang-Gui	Radix Angelicae Sinensis	Angelica sinensis (Oliv.) Diels				
		Chen-Pi	Pericarpium Citri Reticulatae	Citrus reticulate Blanco				
		Sheng-Ma	Radix Cimicifugae	Cimicifuga foetida, L. var., intermedia, Regel				
		Chai-Hu	Radix Bupleuri	Bupleurum chinense DC.				
Liu-Wei-Di-Huang-Wan	Six Ingredient Pill with Rehmannia	Shu-Di-Huang	Radix Rehmanniae	Rehmannia glutinosa Libosch	Deficiency in liver and kidney		1286 (0.02)	7.2
		Shan-Zhu-Yu	Fructus corni officinalis	*Cornus officinalis* Sieb. et Zucc.				
		Shan-Yao	Radix Dioscoreae Oppositae	Dioscorea opposite Thunb.				
		Fu-Ling	Poria	Poria cocos (Schw.) Wolf				
		Mu-Dan-Pi	Cortex Moutan Radicix	Paeonia suffruticosa Andr.				
		Ze-Xie	Rhizoma Alismatis	Alisma orientale (Sam.)Juzep.				
Shao-Fu-Zhu-Yu-Tang	Drive Out Stasis from the Lower Abdomen Decoction	Xiao-Hui-Xiang	Fructus Foenichli Vulgaris	*Foeniculum vulgare* Mill.	Blood stasis and qi stagnation		1030 (0.02)	7.8
		Pao-Jiang	Rhizoma Zingiberis officinales	Zingiber officinale Rosc.				
		Yan-Hu-Suo	Rhizoma Corydalis	Corydalis yanhusuo W. T. Wang				
		Dang-Gui	Radis Angelicae Sinensis	Angelica sinensis (Oliv.) Diels				
		Chuan-Xiong	Rhizoma Chuanxiong	Ligusticum chuanxiong Hort.				
		Mo-Yao	Myrrh	Commiphora molmol, Engi.				
		Rou-Gui	Ramulus Cinnamomi Cassiae	Cinnamomum cassia Blume				
		Chi-Shao	Radix Paeoniae Lactiflorae	Paeonia lactiflora Pall.				
		Pu-Huang	Pollen Typhae	*Typha angustifolia* L.				
		Wu-Ling-Zhi	excrementum Trogopteri Xanthipes	*Trogopterus xanthipes* Milne-Edwards				

*Abbreviation*: *TCM* traditional Chinese medicine, *DUB* dysfunctional uterine bleeding

Frequency indicates the used times of the specific herbal formula, and % indicates the used times of the specific herbal formula over the used times of all herbal formulas for DUB patients

## Discussions

In this study, we found that more than 90% of patients with DUB received TCM treatment. Patients with young age (18–29 y/o) or patients lived in highly urbanized areas were more likely to receive TCM treatment. Of the TCM seekers in our study, more than half of them received both herbal medicine and acupuncture/traumatology. Jia-Wei-Xiao-Yao-San (Bupleurum and Peony Formula) and Yi-Mu-Cao (Herba Leonuri) were the most commonly used TCM formula and single herb, respectively. This was the first population-based cohort study to investigate the TCM utilization patterns among patients with DUB. The analysis of TCM formulas in DUB treatment could provide useful information for further clinical trials and pharmacological investigations.

Our result revealed that patients with young age or lived in highly urbanized areas preferred to receive TCM treatment, which was consistent with the previous studies [16, 17]. TCM is popular among female patients. Our previous study found that patients with uterine fibroid had a high utilization rate of TCM [9]. In this study, we found that large portions of TCM seekers also received conventional treatment. It is possible that patients with better compliance to Western medications would be more likely to use TCM or that the severity of the disease was higher in TCM seekers. Because of the concern over side effects from the conventional treatment, patients with DUB may tend to seek TCM service for a second opinion. More than half of TCM seekers received both herbal medicine and acupuncture. One of the possible reasons is that the DUB patients had more complicated situations that required a combinational treatment of herbal remedies and acupuncture. The complicated situations of patients with DUB as revealed in Table 3 also indicated that they commonly had psychological disorders, anemia, migraine or other systemic disorders.

Of the top ten commonly prescribed formulas identified in our study, Jia-Wei-Xiao-Yao-San and Dang-Gui-Shao-Yao-San share common features to nourish blood, regulate menstrual cycle, and relieve emotional and psychological symptoms clinically. Previous studies found that Jia-Wei-Xiao-Yao-San ameliorated depression in menopausal women through increasing serum TNF-α [18, 19] and Dang-Gui-Shao-Yao-San improved depression-like behaviors in murine model through decreasing central arginine vasopressin [20]. Highly utilization rate of these two formulas may be due to the high incidence of psychological disorders in patients with DUB. Moreover, Dang-Gui-Xiao-Yao-San also exerted analgesic effect on dysmenorrhea through suppression of uterine smooth muscle contractions [21] and corrected luteal phase insufficiency [22]. Gui-Zhi-Fu-Ling-Wan, the formula commonly used to treat menstrual disorders caused by blood stasis, has been demonstrated to exert estrogen-like activity to relieve the symptoms of climacteric disorders [23] and decrease uterine contraction to attenuate dysmenorrhea [24].

Of the ten commonly used single herbs identified in this study, Yan-Hu-Suo (Rhizoma Corydalis) and Xiang-Fu (Rhizoma Cyperi) are traditionally used to treat qi stagnation to relieve pain. Moreover, Yan-Hu-Suo has been used to promote blood circulation, alleviate amenorrhea and dysmenorrhea, and treat puerperal blood stasis [25]. Tetrahydroprotoberberines (THPBs), isolated from Yan-Hu-Suo, was demonstrated to suppress D2 dopamine receptors in the central nervous system to exert analgesic effect [26]. A previous study revealed that Xiang-Fu has estrogen-like and neuroprotective effects in estrogen-deprived mice [27]. The other commonly used herbs in this study, Yi-Mu-Cao (Herba Leonuri) and Dan-Shen (Radix Salviae Miltiorrhizae), were also usually used for the treatment of patients with uterine fibroid [9]. Previous studies demonstrated that leonurine, an alkaloid present in Yi-Mu-Cao, had anti-fibrotic [28], anti-diabetic [29], anti-atherosclerotic [30], and heart protective effects [31] in murine models. Dan-Shen has been demonstrated to enhance the estrogenic effects in ovariectomized rats [32] and stimulate estrogen receptor to exert the effects of anti-oxidative stress [33], anti-inflammation [34] and anti-cancer [35]. Anti-depressive effect of Danshen has been also demonstrated in a rat model [36].

The core pattern of Chinese herbal medicine for DUB patients was the combination of Jia-Wei-Xiao-Yao-San, Xiang-Fu, and Yi-Mu-Cao, which was also the most commonly used combination for premenstrual syndrome [37]. There is no mechanistic study or clinical trials to evaluate the efficacy of this combination. In clinical application according to TCM theory, Jia-Wei-Xiao-Yao-San was developed to treat spleen qi deficiency and liver blood deficiency with heat. Xiang-Fu is used to treat qi stagnation, and Yi-Mu-Cao is used to treat blood stasis. In TCM theory, the combination of Jia-Wei-Xiao-Yao-San, Xiang-Fu, and Yi-Mu-Cao are usually used to supply qi in spleen, supply blood and clean heat in liver, and treat qi stagnation and blood stasis to regulate menstruation. The potential therapeutic efficacy and mechanisms of this combination merit more clinical trials and mechanistic studies.

There are some limitations in our study. The laboratory data and the imaging findings were not available in this database. The differences in disease severity between the TCM seekers and the non-TCM seekers cannot be evaluated. We could only identify the patients who received the conventional drug treatment and surgery in TCM seekers and non-TCM seekers. It has to be noted that DUB requires exclusion diagnostic procedure after clinical and laboratory examination. In the NHIRD datasets, we could identify the specific codes for the laboratory examinations, sonography, and pelvic examination in the datasets. Although we only included those who had at least 2 claims as the DUB patients and further excluded those who were diagnosed as having cervical cancer, endometrial cancer, or ovary cancer within one year of the initial diagnosis of DUB to avoid the selection bias; however, the results of these examinations were not revealed in the datasets. It is also likely that without proper exclusion diagnostic procedures such as clinical and laboratory examination, the number of DUB patients may be exaggerated.

Moreover, herbs purchased at patients’ own expanse beyond the NHI programs were not included in this study since the NHI program only reimburses Chinese herbal medicines manufactured by good manufacturing practice (GMP) -certified pharmaceutical companies in Taiwan. However, because the cost of Chinese herbal products reimbursed by the NHI program is much less than the herbs in the market, the likelihood of purchasing herbs outside of the NHI program is relatively low. In addition, progestin intrauterine device was not reimbursed by the National Health Insurance program until 2015, so we could not identify the patients who used progestin intrauterine device in our study. The other limitation of this study is that the direct efficacy of TCM treatment cannot be evaluated. The compliance to prescriptions was not revealed in the database. These factors should be evaluated in the high-quality, randomized, controlled clinical trials in the future.

## Conclusions

This is the first large-scale population-based study on complementary TCM utilization in patients with DUB. We found that the utilization rate of complementary TCM among patients with DUB is high. The prescription patterns identified in this study could be useful for future clinical studies or pharmacological investigations. Future high-quality, randomized, controlled clinical trials combined with laboratory data may help to determine the efficacy of TCM for DUB patients.

## Abbreviations

DUB: Dysfunctional uterine bleeding
ICD-9-CM: International Classification of Diseases, Ninth Revision, Clinical Modification
NHI: National Health Insurance
NHIRD: National Health Insurance Research Database
NSAIDs: Non-steroidal anti-inflammatory drugs
TCM: Traditional Chinese medicine

## References

[1] Pitkin J (2007). Dysfunctional uterine bleeding. BMJ.

[2] Munro MG, Critchley HO, Broder MS, Fraser IS (2011). Disorders FWGoM: FIGO classification system (PALM-COEIN) for causes of abnormal uterine bleeding in nongravid women of reproductive age. Int J Gynaecol Obstet.

[3] Jensen JT, Lefebvre P, Laliberte F, Sarda SP, Law A, Pocoski J, Duh MS (2012). Cost burden and treatment patterns associated with management of heavy menstrual bleeding. J Women's Health (Larchmt).

[4] Cote I, Jacobs P, Cumming D (2002). Work loss associated with increased menstrual loss in the United States. Obstet Gynecol.

[5] Bradley LD, Gueye NA (2016). The medical management of abnormal uterine bleeding in reproductive-aged women. Am J Obstet Gynecol.

[6] Bongers MY, Mol BW, Brolmann HA (2004). Current treatment of dysfunctional uterine bleeding. Maturitas.

[7] Ray S, Ray A (2016). Non-surgical interventions for treating heavy menstrual bleeding (menorrhagia) in women with bleeding disorders. Cochrane Database Syst Rev.

[8] Zhou J, Qu F (2009). Treating gynaecological disorders with traditional Chinese medicine: a review. Afr J Tradit Complement Altern Med.

[9] Yen HR, Chen YY, Huang TP, Chang TT, Tsao JY, Chen BC, Sun MF (2015). Prescription patterns of Chinese herbal products for patients with uterine fibroid in Taiwan: a nationwide population-based study. J Ethnopharmacol.

[10] National Health Insurance Administration. National Health Insurance Annual Report 2015–2016. Taipie, Taiwan: National Health Insurance Administration, Ministry of Health and Welfare; 2015.

[11] Bensky D, Clavey S, Stoger E (2004). Chinese Herbal Medicine: Materia Medica 3rd Ed.

[12] Scheid V, Bensky D, Ellis A, Barolet R (2009). Chinese herbal medicine: formulas & strategies.

[13] Chan K, Shaw D, Simmonds MS, Leon CJ, Xu Q, Lu A, Sutherland I, Ignatova S, Zhu YP, Verpoorte R (2012). Good practice in reviewing and publishing studies on herbal medicine, with special emphasis on traditional Chinese medicine and Chinese materia medica. J Ethnopharmacol.

[14] Huang MC, Pai FT, Lin CC, Chang CM, Chang HH, Lee YC, Sun MF, Yen HR (2015). Characteristics of traditional Chinese medicine use in patients with rheumatoid arthritis in Taiwan: a nationwide population-based study. J Ethnopharmacol.

[15] Liu C-Y, Hung Y, Chuang Y, Chen Y, Weng W, Liu J, Liang K (2006). Incorporating development stratification of Taiwan townships into sampling design of large scale health interview survey. J Health Manag.

[16] Shih CC, Liao CC, Su YC, Tsai CC, Lin JG (2012). Gender differences in traditional Chinese medicine use among adults in Taiwan. PLoS One.

[17] Pan JC, Tsai YT, Lai JN, Fang RC, Yeh CH (2014). The traditional Chinese medicine prescription pattern of patients with primary dysmenorrhea in Taiwan: a large-scale cross sectional survey. J Ethnopharmacol.

[18] Ushiroyama T, Ikeda A, Sakuma K, Ueki M (2004). Changes in serum tumor necrosis factor (TNF-alpha) with kami-shoyo-san administration in depressed climacteric patients. Am J Chin Med.

[19] Park DM, Kim SH, Park YC, Kang WC, Lee SR, Jung IC: The comparative clinical study of efficacy of Gamisoyo-San (Jiaweixiaoyaosan) on generalized anxiety disorder according to differently manufactured preparations: multicenter, randomized, double blind, placebo controlled trial. J Ethnopharmacol 2014, 158 Pt A:11–17. 10.1016/j.jep.2014.10.024 25456420

[20] Xu F, Peng D, Tao C, Yin D, Kou J, Zhu D, Yu B (2011). Anti-depression effects of Danggui-Shaoyao-san, a fixed combination of traditional Chinese medicine, on depression model in mice and rats. Phytomedicine.

[21] Hsu CS, Yang JK, Yang LL (2006). Effect of "dang-qui-Shao-Yao-san" a Chinese medicinal prescription for dysmenorrhea on uterus contractility in vitro. Phytomedicine.

[22] Usuki S, Higa TN, Soreya K (2002). The improvement of luteal insufficiency in fecund women by tokishakuyakusan treatment. Am J Chin Med.

[23] Namiki T, Sato H, Matsumoto Y, Kakikura H, Ueno K, Chino A, Okamoto H, Hisanaga A, Kaneko A, Kita T (2014). Identification of a predictive biomarker for the beneficial effect of keishibukuryogan, a kampo (Japanese traditional) medicine, on patients with climacteric syndrome. Evid Based Complement Alternat Med.

[24] Sun L, Liu L, Zong S, Wang Z, Zhou J, Xu Z, Ding G, Xiao W, Kou J (2016). Traditional Chinese medicine Guizhi Fuling capsule used for therapy of dysmenorrhea via attenuating uterus contraction. J Ethnopharmacol.

[25] Liao ZG, Liang XL, Zhu JY, Zhao GW, Yang M, Wang GF, Jiang QY, Chen XL (2010). Correlation between synergistic action of radix Angelica Dahurica extracts on analgesic effects of corydalis alkaloid and plasma concentration of dl-THP. J Ethnopharmacol.

[26] Chu H, Jin G, Friedman E, Zhen X (2008). Recent development in studies of tetrahydroprotoberberines: mechanism in antinociception and drug addiction. Cell Mol Neurobiol.

[27] Kim HG, Hong J, Huh Y, Park C, Hwang DS, Choi JH, Oh MS (2013). Cyperi Rhizoma inhibits the 1-methyl-4-phenyl-1,2,3,6-tetrahydropyridine- induced reduction in nigrostriatal dopaminergenic neurons in estrogen-deprived mice. J Ethnopharmacol.

[28] Cheng H, Bo Y, Shen W, Tan J, Jia Z, Xu C, Li F (2015). Leonurine ameliorates kidney fibrosis via suppressing TGF-beta and NF-kappaB signaling pathway in UUO mice. Int Immunopharmacol.

[29] Huang H, Xin H, Liu X, Xu Y, Wen D, Zhang Y, Zhu YZ (2012). Novel anti-diabetic effect of SCM-198 via inhibiting the hepatic NF-kappaB pathway in db/db mice. Biosci Rep.

[30] Zhang Y, Guo W, Wen Y, Xiong Q, Liu H, Wu J, Zou Y, Zhu Y (2012). SCM-198 attenuates early atherosclerotic lesions in hypercholesterolemic rabbits via modulation of the inflammatory and oxidative stress pathways. Atherosclerosis.

[31] Liu X, Pan L, Gong Q, Zhu Y (2010). Leonurine (SCM-198) improves cardiac recovery in rat during chronic infarction. Eur J Pharmacol.

[32] Zhang JM, Li J, Liu EW, Wang H, Fan GW, Wang YF, Zhu Y, Ma SW, Gao XM (2016). Danshen enhanced the estrogenic effects of Qing E formula in ovariectomized rats. BMC Complement Altern Med.

[33] Fan G, Zhu Y, Guo H, Wang X, Wang H, Gao X (2011). Direct vasorelaxation by a novel phytoestrogen tanshinone IIA is mediated by nongenomic action of estrogen receptor through endothelial nitric oxide synthase activation and calcium mobilization. J Cardiovasc Pharmacol.

[34] Fan GW, Gao XM, Wang H, Zhu Y, Zhang J, Hu LM, Su YF, Kang LY, Zhang BL (2009). The anti-inflammatory activities of Tanshinone IIA, an active component of TCM, are mediated by estrogen receptor activation and inhibition of iNOS. J Steroid Biochem Mol Biol.

[35] Nizamutdinova IT, Lee GW, Son KH, Jeon SJ, Kang SS, Kim YS, Lee JH, Seo HG, Chang KC, Kim HJ (2008). Tanshinone I effectively induces apoptosis in estrogen receptor-positive (MCF-7) and estrogen receptor-negative (MDA-MB-231) breast cancer cells. Int J Oncol.

[36] Quan W, Liu F, Zhang Y, Xie C, Wu B, Yin J, Wang L, Zhang W, Zhang X, Wu Q (2015). Antidepressant-like effects of magnesium lithospermate B in a rat model of chronic unpredictable stress. Pharm Biol.

[37] Chen HY, Huang BS, Lin YH, Su IH, Yang SH, Chen JL, Huang JW, Chen YC (2014). Identifying Chinese herbal medicine for premenstrual syndrome: implications from a nationwide database. BMC Complement Altern Med.

